# Constructing a Population-Based Research Database from Routine Maternal Screening Records: A Resource for Studying Alloimmunization in Pregnant Women

**DOI:** 10.1371/journal.pone.0027619

**Published:** 2011-11-30

**Authors:** Brian K. Lee, Alexander Ploner, Zhongxing Zhang, Gunilla Gryfelt, Agneta Wikman, Marie Reilly

**Affiliations:** 1 Department of Epidemiology and Biostatistics, Drexel University School of Public Health, Philadelphia, Pennsylvania, United States of America; 2 Department of Medical Epidemiology and Biostatistics, Karolinska Institutet, Stockholm, Sweden; 3 Department of Clinical Immunology and Transfusion Medicine, Karolinska Institutet and Karolinska University Hospital, Stockholm, Sweden; Institute for Clinical Effectiveness and Health Policy (IECS), Argentina

## Abstract

**Background:**

Although screening for maternal red blood cell antibodies during pregnancy is a standard procedure, the prevalence and clinical consequences of non-anti-D immunization are poorly understood. The objective was to create a national database of maternal antibody screening results that can be linked with population health registers to create a research resource for investigating these issues.

**Study Design and Methods:**

Each birth in the Swedish Medical Birth Register was uniquely identified and linked to the text stored in routine maternal antibody screening records in the time window from 9 months prior to 2 weeks after the delivery date. These text records were subjected to a computerized search for specific antibodies using regular expressions. To illustrate the research potential of the resulting database, selected antibody prevalence rates are presented as tables and figures, and the complete data (from more than 60 specific antibodies) presented as online moving graphical displays.

**Results:**

More than one million (1,191,761) births with valid screening information from 1982–2002 constitute the study population. Computerized coverage of screening increased steadily over time and varied by region as electronic records were adopted. To ensure data quality, we restricted analysis to birth records in areas and years with a sustained coverage of at least 80%, representing 920,903 births from 572,626 mothers in 17 of the 24 counties in Sweden. During the study period, non-anti-D and anti-D antibodies occurred in 76.8/10,000 and 14.1/10,000 pregnancies respectively, with marked differences between specific antibodies over time.

**Conclusion:**

This work demonstrates the feasibility of creating a nationally representative research database from the routine maternal antibody screening records from an extended calendar period. By linkage with population registers of maternal and child health, such data are a valuable resource for addressing important clinical questions, such as the etiological significance of non-anti-D antibodies.

## Introduction

The screening of pregnant women for the presence of red blood cell (RBC) antibodies is a standard prenatal procedure in developed countries. The primary purpose of this screening is the prevention of hemolytic disease of the fetus and newborn (HDFN), which can result from maternal RBC antibodies crossing the placental barrier into fetal circulation and attacking fetal RBCs [1]. Anti-rhesus D (or simply, anti-D) antibody has long been recognized to be responsible for most cases of HDFN [2]. Due to screening for anti-D and associated immunoprophylactic measures implemented since the 1970's in Europe and the U.S., the prevalence of anti-D antibodies in pregnant women has decreased from approximately 10% in RhD negative women to a current level of 0.1 to 2%, depending on whether routine antenatal Rh-prophylaxis is used [3]. In addition to anti-D antibody, there are more than 50 antibodies reported to be associated with HDFN [4]. Some of these RBC antibodies, such as anti-c and anti-K, can cause severe disease in the fetus or newborn, while others such as anti-C, -E, -e, -Fya, -Fyb, -Jka, Jkb, -M, -N, -S, and -s are considered to be non-aggressive but nonetheless are carefully monitored. Others such as anti-Lea, -Leb, -P1, and -A1 are considered clinically insignificant.

Despite widespread routine screening for maternal antibodies in recent decades and the use of electronic databases for managing the screening program and laboratory results, there has been no effort to our knowledge to construct national databases from these data for research purposes. In contrast to other areas of health service (notably cancer diagnosis and treatment) where standardized population registers are used to study national trends, studies of alloimmunization during pregnancy typically represent a limited geographical area and/or time period [5]–[7].The focus of published work has been anti-D immunization and the associated prophylaxis routines that have resulted in a significant reduction in HDFN. Research into the distribution, determinants and clinical consequences of non-anti-D RBC antibodies has been hampered by the lack of large population databases, as these antibodies have low prevalence and thus require a study population larger than what is typically available. However, as anti-D antibody prevalence declines, these non-anti-D maternal antibodies are associated with an increasing proportion of cases of erythrocyte immunization and HDFN and warrant careful studies of their prevalence and potential risks for fetal and newborn health. The objective of our work was to investigate the feasibility of constructing a national research database for addressing such questions using the computerized routine maternal screening records of an entire population over an extended calendar period.

This manuscript describes the compilation of a database from the routine blood typing and antibody screening results for over one million pregnancies in Sweden. We illustrate the research potential of such data by presenting the time trends in nationwide prevalence rates of various RBC antibodies over a 20-year calendar period.

## Methods

### Ethics Statement

This research was approved by the Stockholm Regional Ethics committee (Diary number 2008/672-32). Informed consent was not obtained since all personal information was made anonymous to investigators prior to analysis in accordance with Swedish law.

### Swedish maternal screening program

For a majority of pregnant women in Sweden, ABO/RhD typing and RBC antibody screening of serum or plasma samples occur at the first prenatal appointment between the 8th and 10th weeks. In recent years, most RhD positive women without irregular antibodies are screened just once, while RhD negative women are retested at the 25th and 35th weeks. For positive antibody screening test results, laboratory identification of the antibodies and quantification of antibody titers is subsequently performed at the regional blood centers. Because prenatal care in Sweden is provided at no cost for all women, compliance with screening guidelines is expected to be high.

The technology used for antibody screening has changed over time. In the early years of this study the maternal antibody screening was done using a manual indirect antiglobulin test (IAT) in tubes and against enzyme treated RBC. From the mid-1980's, low ionic strength solution (LISS) was used as the RBC suspension medium, replacing phosphate buffered saline. In 1989–1990, gelcard technology was introduced in the country. From 1993–1994, enzyme screening as first line screening was abandoned, leaving gel IAT as the routine screening method in most laboratories. Procedures with solid phase techniques were introduced from the mid-1990s and fully automated systems with microcolumn technology were introduced from around 2000.

### Data sources

Maternal antibody screening records were extracted from the Scandinavian Donations and Transfusions (SCANDAT) database which contains records on blood donation, transfusion, and laboratory tests for any person who was registered in any of the regional blood center databases in Sweden since 1968. SCANDAT has been described in detail elsewhere [8]. Briefly, computerized data were obtained from all regional blood centers in Sweden and used to create a relational database, where the individuals were linked to national population and health registers using their unique ten-digit national identity number. Following confidentiality requirements, the cleaned and validated data were permanently de-identified by replacing each person's national identity number with a random study number.

The Swedish Medical Birth Register (SMBR) contains data on all births in Sweden since 1973, including mother's age, country of birth, county of residence and parity, and delivery and infant details including singleton and multiple births and stillbirths, infant sex, and neonatal diagnoses coded using the ICD classification system. The Swedish Medical Birth Register includes 98–99% of all births in Sweden and the quality of the data is high [9]. At the time of de-identification of the SCANDAT database, it included records from the SMBR from 1st January 1982 through 31st December 2002.

### Data Extraction

The unit of analysis in this manuscript is a birth – each individual infant born, whether in a single or multiple delivery, and including live births and still births. Each birth was uniquely identified based on mother's study number, date of birth, parity, and birth order. For each birth in the SMBR from 1982–2002, maternal antibody screening records were extracted from SCANDAT in the time window from 9 months prior to 2 weeks after the delivery date.

Because laboratory results were stored in free text format, a computerized search was undertaken for text corresponding to specific antibodies. Since a woman must first screen positive with a non-specific test in order to be subjected to testing for specific antibodies, these text data are confirmatory tests for the presence of antibodies, thereby reducing the likelihood of a false positive test result. The search list of antibodies was compiled by examining the more structured text data and from published reports of known antibodies. The computerized search used regular expressions, a flexible software means of identifying and extracting specified text patterns that has been used to perform automated informatics tasks with free-text clinical records [10]. Since our data were stored as an Oracle database, we used POSIX regular expression syntax. The regular expressions accounted for differences in notation, capitalisation, spacing and use of punctuation marks (see **Table S1**).

#### Validation

To validate the accuracy and completeness of the computerized search, we visually examined all the text information from a sample of 2374 laboratory records, representing all pregnancy-related screening and antibody tests for 200 randomly chosen pregnancies, 100 of which were selected at random from those defined as antibody positive by our computer code and 100 selected from those defined as negative. Using this subsample, our validation calculated three measures of the performance of the automated search algorithm: positive predictive value (PPV) for women identified as antibody positive; negative predictive value (NPV) for women identified as antibody negative; and antibody identification accuracy of the antibodies in women who were truly antibody positive. For all 200 women, human examination verified that the computerized search had correctly classified whether or not any antibodies were present, yielding a PPV and NPV of 100%. Thus based on a Binomial distribution, an approximate 95% confidence interval for the PPV and NPV of our search algorithm is (0, 3%). Among the 100 positive women, one specific antibody was missed and one misspelled laboratory result entry from the technician was misclassified as an antibody, which errors would have resulted in a false negative and false positive test respectively if these were the only specific antibodies present. The 100 antibody positive women had 108 specific antibody terms found by the search algorithm, of which 107 were verified. Thus the accuracy of the algorithm in recognizing specific antibody search terms within our validation subsample is 0.99 (95% confidence interval: 0.95, 1.00).

### Illustrative data analysis and visualization

To illustrate the research potential of the database, we present selected antibody prevalences in this manuscript as tables and figures, and the complete data (from more than 60 specific antibodies) are available online as moving graphical displays, which are becoming a prominent and effective tool for data presentation [11].

### Software

All data preparation steps used SAS version 9.2 (SAS Institute, Cary, NC, USA) and its SQL capabilities. Software for data visualization and analysis included SAS, R (R Foundation for Statistical Computing, Vienna, Austria; www-r-project.org) and the Motion Chart application from Google (Google Visualization API, Mountain View, CA, USA).

## Results

### Definition of the study population

**Figure 1:**
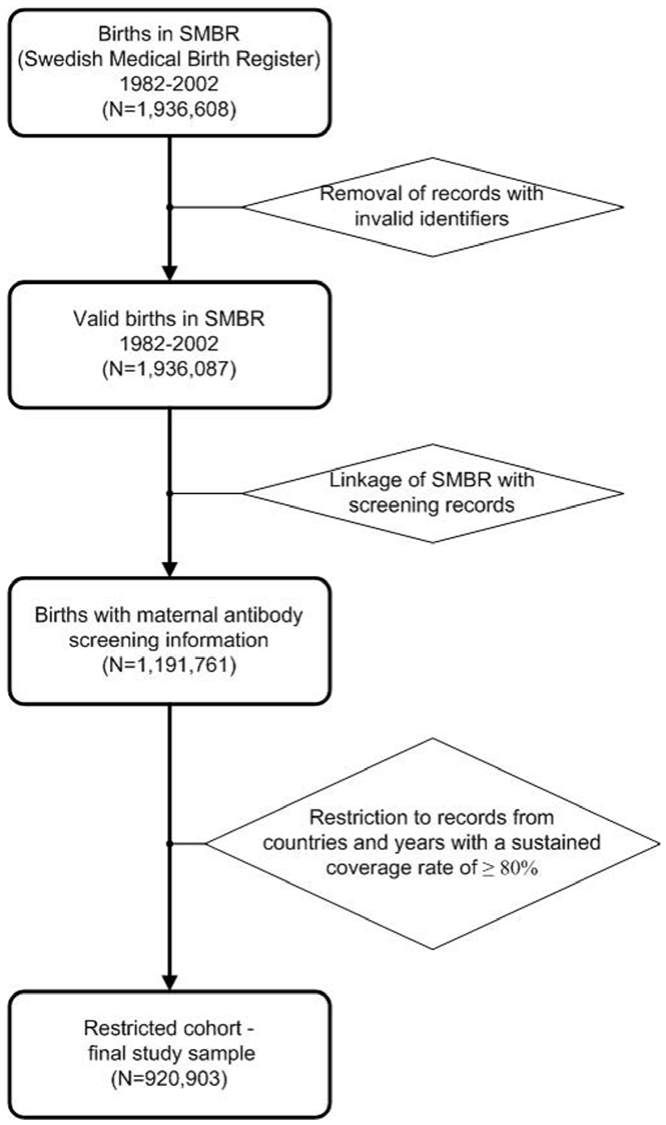
Descriptive schematic of the extraction of the final study sample.

**Figure 2:**
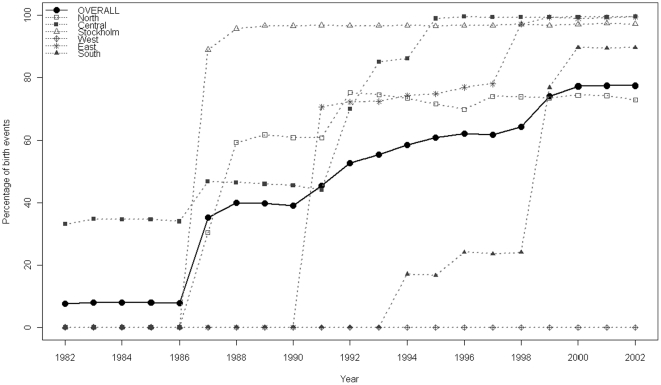
Percentage of Swedish births from 1982–2002, stratified by geographical region, that are included in the study population.

The database contains information on 1,936,608 birth events occurring from 1982–2002. After data cleaning and removal of records with invalid identifiers, a total of 1,936,087 births from 1,029,008 individual women were available. A total of 1,191,761 births (i.e. 61.6%) could be linked with valid maternal screening records (see **Figure 1**). The proportion of births with valid screening data increased steadily over time (from 7.5% in 1982 to 77.5% in 2002) as electronic medical records became more widespread. Regional variations in data coverage were largely attributable to different dates of introduction of computerization (see **Figure 2**). Coverage rates in some centres were also affected by technical or administrative issues: for example, data from several laboratories were excluded because of systematically missing laboratory dates, while administrative difficulties prevented the acquisition of maternal screening data from some laboratories.

A birth was deemed antibody-positive for a given antibody if the antibody was mentioned in at least one of the maternal antibody records associated with the birth. For anti-D antibody, a birth was only deemed positive if the positive finding was not suspected to be due to RhD prophylaxis (i.e., if no search terms indicating prophylaxis administration were found).

To limit potential errors due to inconsistent data reporting practices and to minimize any potential reporting bias in favor of positive antibody findings, we restricted analysis to records from women who gave birth in counties and years with a sustained data coverage rate of at least 80%. For example, although data from Stockholm County were available from 1982, the coverage only reached the 80% threshold in 1987, increasing to 98–99% in subsequent years (see Figure 2). From 1982 to 1986, only two counties contributing 28,829 births (7.8% of the total births in the period) were included. As more areas of Sweden reached sufficient data coverage to be included, the proportion of total births represented in the study sample increased to 40.2% in 1987–1991, 57.7% in 1992–1996, and 77.5% in 2002 (see Figure 2). It is worth noting that one region of Sweden, accounting for 121,303 birth events with antibody data, did not meet the inclusion criteria at any time and were thus excluded from analysis. The coverage rate for all regions included in our analysis was 93.7%.

### Description of the study population

From 1 January 1982 to 31 December 2002, 920,903 births (representing 17 of the 24 counties of Sweden and 47.6% of the 1,936,087 total valid births in the period) from 572,626 mothers satisfied the inclusion criteria. The characteristics of these births are summarized in **Table 1**. Maternal age at delivery increased from an average of 28.0 years from 1982–1986 to 30.1 years from 1997–2002. The percentage of RhD negative women was consistent (14–15%) across all years.

**Table 1:** Characteristics of births in Sweden included in maternal red blood cell antibody prevalence rates, 1982–2002.

Characteristic	1982–1986	1987–1991	1992–1996	1997–2002	Overall, 1982–2002
**Births, N**	28,829	205,809	303,975	382,290	920,903
**Individual women, N**	23,673	164,287	244,412	301,342	572,626*
**Maternal age (years) at delivery, mean (SD)**	28.5 (5.1)	28.9 (5.1)	29.3 (5.0)	30.1 (5.0)	29.5 (5.1)
**Maternal RhD bloodgroup, % births**					
RhD+	85.1	84.5	85.1	85.2	85.0
A+	39.7	38.5	38.6	38.2	38.5
B+	10.0	9.9	10.1	10.3	10.2
AB+	4.7	4.7	4.6	4.5	4.6
O+	30.7	31.3	31.8	32.2	31.8
RhD−	14.8	14.5	14.6	14.6	14.6
A−	6.8	6.7	6.8	6.7	6.7
B−	1.7	1.6	1.6	1.7	1.6
AB−	0.8	0.8	0.8	0.8	0.8
O−	5.4	5.3	5.4	5.5	5.4
Unknown	0.1	1.0	0.2	0.1	0.4
**Parity, mean**	1.9	1.9	2.0	1.9	1.9
**Male child, %**	51.3	51.3	51.2	51.4	51.3
**Mother's country of birth, % births**					
Sweden	86.6	84.2	84.3	82.5	83.6
Other Nordic country	7.6	6.2	3.9	2.4	3.9
Other country	5.6	9.6	11.8	15.1	12.5
Unknown	0.3	0.1	0.1	0.1	0.1

*Note: Because an individual woman can give birth in more than one time period, the number of women overall from 1982–2002 does not equal the row total.

### Frequency of antibodies

From 1982–2002, a total of 8,141 births (0.88% of all births nationwide with screening information) from 6,428 women (1.1%) were associated with at least one maternal RBC antibody. **Table 2** presents antibodies that had a prevalence of at least 1 in 10,000 in our data, and the rarer antibodies are presented as supplementary information in **Table S2**. Interactive graphics of all antibodies and prevalences across each year are presented in **Figure S1**. Similar graphical displays examined by region indicated a stabilizing of antibody prevalences from the time a sustained coverage of at least 80% was achieved. Rhesus system antibodies were the most common (33.3 per 10,000 births), followed by Lewis system (17.6/10,000), MNS system (9.4/10,000), and Kell system antibodies (7.0/10,000). Of the 8,141 births that were antibody-positive, 15.9% were positive for anti-D. The most frequently occurring non-anti-D antibodies were anti-Lea (13.9/10,000), anti-E (13.0/10,000), anti-M (8.0/10,000), and anti-K (5.8/10,000). Multiple antibodies were detected in 877 births (10.8% of all antibody-positive births) from 732 women. The most frequently found combinations of antibodies are displayed in **Table 3**, of which the combination of anti-C and anti-Cw was the most frequent (1.4% of antibody-positive births), followed by anti-c and anti-E (1.3%), anti-Lea and anti-Leb (1.1%), and anti-D and anti-C (1.0%).

**Table 2:** Maternal red blood cell antibodies with a prevalence of at least 1 in10,000 in 920,903 births in Sweden from 1982–2002.

	*Per 10,000 births*						
					Overall		Percent of
	1982–1986	1987–1991	1992–1996	1997–2002	1982–2002	Count	Ab+ births
**Any antibody**	53.1	81.4	102.3	83.8	88.4	8141	100
**Non-anti-D antibodies**	41.3	70.6	90.6	71.9	76.8	7072	86.9
**Rh system**	18.4	28.9	36.8	33.9	33.3	3063	37.6
anti-D	12.1	12.3	14.8	14.6	14.1	1296	15.9
anti-E	2.8	10.7	15.7	12.9	13	1198	14.7
anti-C	0.4	3.5	4.9	5.1	4.5	418	5.1
anti-c	1	3.9	4.9	4.7	4.5	411	5
anti-Cw	0	1.8	1.9	2.1	1.9	174	2.1
**Lewis system**	26.4	23.8	19.7	11.8	17.6	1616	19.9
anti-Lea	19.8	19.1	15.7	9.3	13.9	1283	15.8
anti-Leb	6.6	6	5.5	3.2	4.7	435	5.3
**MNS system**	0.7	4.4	9.4	12.7	9.3	861	10.6
anti-M	0.7	3.6	8.2	10.8	8	738	9.1
**Kell system**	4.2	6.9	6.7	7.5	7	644	7.9
anti-K	4.2	6.3	6.2	5.4	5.8	534	6.6
**P system**	0	3.9	6.6	3.6	4.6	420	5.2
anti-P1	0	3.9	6.6	3.5	4.5	415	5.1
**Duffy system**	0	1.9	2.6	2.2	2.2	205	2.5
anti-Fya	0	1.8	2.4	2.1	2	189	2.3
**Kidd system**	0.7	1.4	1.9	2.7	2.1	191	2.4
anti-Jka	0.7	1.3	1.7	2.2	1.8	165	2
**Lutheran system**	0.4	0.9	0.6	1.5	1.1	97	1.2
anti-Lua	0.4	0.9	0.6	1.5	1	95	1.2
**Other**							
Unspecified antibodies	0.4	4.2	10.7	3.7	6.0	554	6.8
anti-A1	0.7	2.9	2.8	1.8	2.4	217	2.7
anti-Bg	0	1	3	3.1	2.5	229	2.8

**Table 3:** Most frequently occurring combinations of maternal red blood cell antibodies in 920,903 births in Sweden from 1982–2002: total number of births and percentage of antibody-positive births.

Antibody combination	Count	Percent of Ab+ births
Anti-C, Anti-Cw	116	1.4
Anti-E, Anti-c	105	1.3
Anti-Lea, Anti-Leb	93	1.1
Anti-D, Anti-C	82	1.0
Anti-D, Anti-E	38	0.5
Anti-D, Anti-C, Anti-E	36	0.4
Anti-C, Anti-e	17	0.2
Anti-K, Anti-E	16	0.2

### Temporal trends

**Figure 3:**
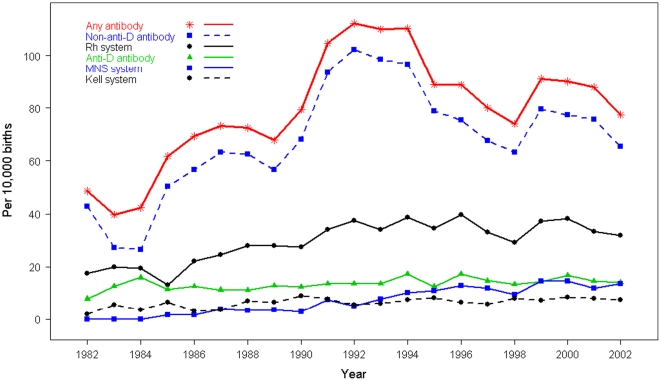
Selected maternal red blood cell antibody prevalence rates in Sweden, 1982–2002.

Maternal RBC antibody prevalence estimates in 1982-1986 are lower (range 39.7–69.4/10,000 births) than in subsequent years (1987–2002: range 67.9–112.3/10,000) (**Figure 3**). Anti-D antibody prevalence rates remained constant from 1987–2002, ranging from 11.2 to 17.2/10,000 births. Non-anti-D antibody prevalences were constant from 1987 (63.4/10,000 births) until a sharp increase between 1990 (68.3/10,000) and 1991 (93.6/10,000). This elevated non-anti-D antibody prevalence was sustained until 1994 (96.6/10,000) but then decreased to fluctuate around the 2002 rate of 65.6/10,000. Prevalence rates for the clinically significant antibodies anti-c and anti-K remained constant across the entire study period (overall 1982–2002 rates of 4.5 and 5.8/10,000 births, respectively). Anti-E antibody prevalences fluctuated from a 1987 rate of 8.3/10,000 to a 2000 rate of 16.6/10,000 for an overall 1982–2002 rate of 13.0/10,000.

## Discussion

We have demonstrated the feasibility of using routine maternal antibody screening data to establish a large population-based research database covering over 20 years of screening activity on a well-defined population of pregnant women. The research potential of the database is illustrated by presenting the prevalence rates for a large number of specific maternal antibodies over a long calendar period. To our knowledge, this provides the first comprehensive assessment of such a wide range of RBC antibodies for a well-defined population, as heretofore sample sizes have been insufficient to study antibodies with low prevalence.

The data provided a number of interesting insights into the alloimmunization in our population. Even in the age of prophylactic treatment with immune globulin, immunization with anti-D antibody in pregnant women still occurs. The observed prevalence of anti-D immunization in this nationwide cohort from 1982–2002 was 14.1/10,000 births, comparable with previous regional estimates of 14.2/10,000 in 1980–1991 [6] and 12.6/10,000 in 1983–1989 [12]. Although we limited our analysis to data from regions that had achieved a sustained high coverage rate, similar prevalence estimates were obtained from all available data, suggesting that reporting bias is not a serious concern. Immunization with non-anti-D antibodies was much more common than with anti-D, occurring at over 5 times the frequency. Interestingly, the rate of anti-D immunization was fairly constant across the years, in contrast to the sharp increase in non-anti-D antibody immunization between 1990 and 1991, though these trends may reflect in part the changes in screening technology during that time. The absence of information concerning technologies used is a feature of many registers that needs to be considered in data analysis. Where information can be obtained from the laboratories conducting the assays, this can be included as a potential confounder in epidemiological analysis. Since such information is often unavailable or difficult to obtain, the changes in sensitivity and specificity due to technical progress are more commonly dealt with by careful statistical modeling of calendar time (and perhaps regional) effects.

The purpose of our analysis was to illustrate the potential of good quality data extracted from routine clinical laboratory records for a given population, and not to generalize our findings to other populations. Antibody prevalence rates are known to depend on a number of population-specific factors, including underlying antigen genotype frequencies and histories of sensitization. For example, while samples in the U.S. [13], Canada [14], [15], England [5], the Netherlands [16], and Norway [17] report maternal anti-D prevalences that are similar to our estimates (between 8–23/10,000 births), a Kuwaiti sample reported a prevalence of 522/10,000, a rate that may be partially explained by the high local fertility rate [18]. Prevalence rates are also dependent on trends in clinical practice and screening technologies, so that observed changes over time are at least partly due to different routines and technologies used. For example, in the mid-1980's LISS/IAT replaced saline IAT as the screening method in Sweden, thereby increasing the sensitivity of antibody detection. The introduction of the gel card technology in the early 1990's increased the sensitivity for many clinically significant antibodies (e.g., Rhesus antibodies, Kidd antibodies and Duffy antibodies) while fewer Lewis antibodies were detected. When enzyme screening was abandoned in 1994, unspecific reactions, often reported as unspecified antibodies, decreased.

The database described in this manuscript is constructed from the available data in the SCANDAT donation and transfusion database. Due to ethical requirements, the SCANDAT database was anonymised after compilation and it was thus not possible to perform further linkages to the routine maternal screening data that accumulated since 2002. However, the construction of our maternal RBC antibody database was a “proof of principle” exercise whose feasibility provides important support for future data linkages to all available population screening records.

For the period 1982–2002, our database represents a large proportion of all births in Sweden, although there are a number of shortcomings. As mentioned previously, administrative difficulties prevented the acquisition of data from several centers of sufficiently high quality to merit inclusion in the present study. In addition, data entry practices differ by laboratory and over time so that not all laboratory results were computerized in a fashion that was usable (e.g., missing sample identifiers and laboratory test dates). The highlighting of quality control issues is an important outcome of our work and can help to improve the quality of the source data and in turn the quality of data obtained from future linkages.

The volume of maternal antibody records in the database and the storage of this information as free text required the computerized extraction of data similar to previous investigations of blood bank data for central Sweden [6]. The performance of these automated approaches is dependent on the capabilities of the extraction method as well as the structure and consistency of the free text records. The computerized search process yielded very high agreement with human abstraction in a validation sample of over 2000 records for 200 pregnant women, with high PPV, NPV and accuracy in identifying antibodies. PPV and NPV for nonspecific antibody status are important due to the increased monitoring for women who test positive for any antibody (regardless of the antibody). Similarly, identification accuracy is important to ensure that specific antibodies which can have different medical consequences are correctly reflected in the database. A limitation of the current version of the database is that only the presence or absence of antibodies is characterized: titer information is present for some records but the lack of a consistent data structure presents a challenge in extracting this information.

The building of a maternal screening database such as ours is the first step in investigating a number of important research questions. By linkage with population registers of maternal and child health, clinically relevant questions can be investigated, such as identification of risk factors and important confounders (notably history of transfusion) for immunization; etiological significance of various antibodies for fetal and pediatric outcomes including stillbirth, prematurity, infant death, hyperbilirubinemia, and anemia. In particular, investigation of the clinical relevance of non anti-D antibodies has the potential to inform policies for the management of pregnant women with these antibodies: the current recommendation of the American College of Obstetricians and Gynecologists is to follow the same care guidelines as for Rhesus alloimmunization, although there is limited evidence to support this practice [19], [20]. In addition, linkage with other population registers would enable the study of associations between RBC antibodies and subsequent health outcomes in women such as hospitalizations, overall mortality, cancer and immune disorders.

## Supporting Information

Figure S1 **(a)** – Changes in prevalence of maternal antibodies with bubbles proportional to numbers detected. **(b)** – Trends in prevalence of specific antibodies (chosen by the viewer), displayed as line graphs. **(c)** – Changes in prevalence of 4 specific antibodies (anti-E,K,M,c) displayed as moving bar-charts. **(d)** – Trends in prevalence of antibody systems displayed as line graphs connecting bubbles proportional to numbers detected.
(HTML)

Table S1 Regular expressions used to identify 64 specific antibodies.
(DOC)

Table S2 Maternal red blood cell antibodies with a prevalence of less than 1 in10,000 in 920,903 births in Sweden from 1982–2002.
(DOCX)
