# Supplementary material for: Constructing a Population-Based Research Database from Routine Maternal Screening Records: A Resource for Studying Alloimmunization in Pregnant Women
Source: PLoS One. 2011 Nov 30;6(11):e27619. doi: 10.1371/journal.pone.0027619 (PMC3227597; doi:10.1371/journal.pone.0027619)
Supplement: Figure S1 — (a) – Changes in prevalence of maternal antibodies with bubbles proportional to numbers detected. (b) – Trends in prevalence of specific antibodies (chosen by the viewer), displayed as line graphs. (c) – Changes in prevalence of 4 specific antibodies (anti-E,K,M,c) displayed as moving bar-charts. (d) – Trends in prevalence of antibody systems displayed as line graphs connecting bubbles proportional to numbers detected. (HTML) [file pone.0027619.s001.html]

  
  
  

**Epidemiology of anti-D and non-anti-D** 

**maternal red blood cell antibodies in Sweden:** 
  
  

**- prevalence rates (per 10,000 births) and counts (births)** 
  
  


---

  

*Authors: Brian K. Lee, Alexander Ploner, Zhongxing Zhang,
Gunilla Gryfelt, Agneta Wikman, Marie Reilly* 
  
  
  
  
  

- Charts require an internet connection and a browser with the 
Adobe Flash plugin
  

- Please experiment with any of
these figures to create your own dynamic display. For example, the
Y-axis can display antibody counts or rates. Also, motion charts, bar
charts, and scatterplots are selectable by clicking on the upper right
corner of the figures.


  
  

(a) - Prevalence of maternal RBC (red blood cell) antibodies in Sweden 1982-2002. 

\* Please click "Play" button in the left lower corner to see changes over time, and please hover your mouse over any column of interest and the name of the antibody will be displayed aside it. 
  
  
  
  

(b) - Prevalence of specific maternal RBC antibodies in Sweden 1982-2002. 

\* Please choose any antibodies of interest in the right column 
  
  
  
  

(c) - Prevalence of specific maternal RBC antibodies in Sweden 1982-2002.

\* Please choose antibodies of interest in the right column then click "Play" button, you can also zoom-in by dragging your mouse or zoom-out in the right lower corner 
  
  
  
  

(d) - Prevalence of maternal RBC antibody systems in Sweden 1982-2002. 

\* Please choose antibodies of interest in the right column then click "Play" button
