# Supplementary material for: Constructing a Population-Based Research Database from Routine Maternal Screening Records: A Resource for Studying Alloimmunization in Pregnant Women
Source: PLoS One. 2011 Nov 30;6(11):e27619. doi: 10.1371/journal.pone.0027619 (PMC3227597; doi:10.1371/journal.pone.0027619)
Supplement: Table S1 — Regular expressions used to identify 64 specific antibodies. (DOC) [file pone.0027619.s002.doc]

**Table S1: Regular expressions used to identify 64 specific antibodies.**

| **ANTIBODY** | **REGULAR EXPRESSION** | **MATCHING ANTIBODIES AND SYNONYMS** |
| --- | --- | --- |
| anti-A1 | [aA]nti[[:space:]]*[-:][[:space:]]*A1 | anti-A1 |
| anti-Bg | [aA]nti[[:space:]]*[-:][[:space:]]*B(g|ga|g\(a\)|gb|g\(b\)) | anti-Bg(a), anti-Bg(b), anti-Bga, anti-Bgb, anti-Bg |
| anti-C | [^[:alnum:]][aA]nti[[:space:]]*[-:][[:space:]]*C[^[:alnum:]] | anti-C |
| anti-c | [aA]nti[[:space:]]*[-:][[:space:]]*c[^[:alnum:]] | anti-c |
| anti-Ce | [aA]nti[[:space:]]*[-:][[:space:]]*Ce | anti-Ce |
| anti-cE | [aA]nti[[:space:]]*[-:][[:space:]]*cE | anti-cE |
| anti-ce | [aA]nti[[:space:]]*[-:][[:space:]]*(ce|f) | anti-ce, anti-f |
| anti-CE | [aA]nti[[:space:]]*[-:][[:space:]]*CE | anti-CE |
| anti-Ch | [aA]nti[[:space:]]*[-:][[:space:]]*C(h|ha|h\(a\)|hido) | anti-Ch, anti-Ch(a), anti-Chido, anti-Cha |
| anti-Coa | [aA]nti[[:space:]]*[-:][[:space:]]*Co(a|\(a\)) | anti-Coa, anti-Co(a) |
| anti-Cob | [aA]nti[[:space:]]*[-:][[:space:]]*Co(b|\(b\)) | anti-Cob, anti-Co(b) |
| anti-CSa | [aA]nti[[:space:]]*[-:][[:space:]]*C[sS][aA] | anti-Csa, anti-CSa, anti-CSA, anti-CsA |
| anti-Cw | [aA]nti[[:space:]]*[-:][[:space:]]*(C[wW]|C\([wW]\)|R8) | anti-C(W), anti-C(w), anti-CW, anti-Cw, anti-R8 |
| anti-D | [^[:alnum:]][aA]nti[[:space:]]*[-:][[:space:]]*(D|RH|Rh) | anti-D, anti-RH, anti-Rh |
| anti-E | [^[:alnum:]][aA]nti[[:space:]]*[-:][[:space:]]*E[^[:alnum:]] | anti-E |
| anti-e | [^[:alnum:]][aA]nti[[:space:]]*[-:][[:space:]]*e[^[:alnum:]] | anti-e |
| anti-Ena | [aA]nti[[:space:]]*[-:][[:space:]]*E(n|na|n\(a\)) | anti-En, anti-Ena, anti-En(a) |
| anti-Fy3 | [aA]nti[[:space:]]*[-:][[:space:]]*Fy3 | anti-Fy3 |
| anti-Fya | [aA]nti[[:space:]]*[-:][[:space:]]*Fy(a|\(a\)) | anti-Fya, anti-Fy(a) |
| anti-Fyb | [aA]nti[[:space:]]*[-:][[:space:]]*Fy(b|\(b\)) | anti-Fyb, anti-Fy(b) |
| anti-G | [aA]nti[[:space:]]*[-:][[:space:]]*G[^[:alnum:]] | anti-G |
| anti-Ge | [aA]nti[[:space:]]*[-:][[:space:]]*Ge | anti-Ge |
| anti-H | [aA]nti[[:space:]]*[-:][[:space:]]*(H|HA)[^[:alnum:]] | anti-H, anti-HA |
| anti-HI | [aA]nti[[:space:]]*[-:][[:space:]]*HI | anti-HI |
| anti-I | [aA]nti[[:space:]]*[-:][[:space:]]*I([^[:alnum:]]|[Aa]) | anti-I, anti-IA, anti-Ia |
| anti-i | [aA]nti[[:space:]]*[-:][[:space:]]*i([^[:alnum:]]|a) | anti-i, anti-ia |
| anti-IH | [aA]nti[[:space:]]*[-:][[:space:]]*IH | anti-IH |
| anti-JK3 | [aA]nti[[:space:]]*[-:][[:space:]]*Jk3 | anti-Jk3 |
| anti-Jka | [aA]nti[[:space:]]*[-:][[:space:]]*Jk(a|\(a\)) | anti-Jk(a), anti-Jka |
| anti-Jkb | [aA]nti[[:space:]]*[-:][[:space:]]*Jk(b|\(b\)) | anti-Jk(b), anti-Jkb |
| anti-JMH | [aA]nti[[:space:]]*[-:][[:space:]]*JMH | anti-JMH |
| anti-Jra | [aA]nti[[:space:]]*[-:][[:space:]]*Jr(a|\(a\)) | anti-Jr(a), anti-Jra |
| anti-Jsa | [aA]nti[[:space:]]*[-:][[:space:]]*(Jsa|Js\(a\)|K6) | anti-Js(a), anti-Jsa, anti-K6 |
| anti-Jsb | [aA]nti[[:space:]]*[-:][[:space:]]*(Jsb|Js\(b\)|K7) | anti-Js(b), anti-Jsb, anti-K7 |
| anti-K | [aA]nti[[:space:]]*[-:][[:space:]]*K([^[:alnum:]]|ell|1) | anti-K, anti-Kell, anti-K1 |
| anti-k | [aA]nti[[:space:]]*[-:][[:space:]]*(k|K2) | anti-k, anti-K2 |
| anti-Kna | [aA]nti[[:space:]]*[-:][[:space:]]*Kn(a|\(a\)) | anti-Kn(a), anti-Kna |
| anti-Kpa | [aA]nti[[:space:]]*[-:][[:space:]]*(Kpa|Kp\(a\)|K3) | anti-Kp(a), anti-Kpa, anti-K3 |
| anti-Kpb | [aA]nti[[:space:]]*[-:][[:space:]]*(Kpb|Kp\(b\)|K4) | anti-Kp(b), anti-Kpb, anti-K4 |
| anti-Ku | [aA]nti[[:space:]]*[-:][[:space:]]*(Ku|K5) | anti-Ku, anti-K5 |
| anti-Lan | [aA]nti[[:space:]]*[-:][[:space:]]*[lL][aA][nN] | anti-lan, anti-Lan, anti-LAN |
| anti-Lea | [aA]nti[[:space:]]*[-:][[:space:]]*Le(a|\(a\)) | anti-Lea, anti-Le(a) |
| anti-Leb | [aA]nti[[:space:]]*[-:][[:space:]]*Le(b|\(b\)) | anti-Leb, anti-Le(b) |
| anti-Lua | [aA]nti[[:space:]]*[-:][[:space:]]*Lu(a|\(a\)) | anti-Lua, anti-Lu(a) |
| anti-Lub | [aA]nti[[:space:]]*[-:][[:space:]]*Lu(b|\(b\)) | anti-Lub, anti-Lu(b) |
| anti-LW | [aA]nti[[:space:]]*[-:][[:space:]]*L[wW] | anti-LW, anti-Lw |
| anti-M | [^[:alnum:]][aA]nti[[:space:]]*[-:][[:space:]]*M[^[:alnum:]] | anti-M |
| anti-McCa | [aA]nti[[:space:]]*[-:][[:space:]]*McC(a|\(a\)) | anti-McCa, anti-McC(a) |
| anti-N | [aA]nti[[:space:]]*[-:][[:space:]]*N | anti-N |
| anti-P1 | [aA]nti[[:space:]]*[-:][[:space:]]*P([^[:alnum:]]|1) | anti-P, anti-P1 |
| anti-PP1Pk | [aA]nti[[:space:]]*[-:][[:space:]]*(PP1Pk|Tj(a|\(a\))|T[Ii]j) | anti-PP1Pk, anti-Tja, anti-Tj(a), anti-Tij |
| anti-Pk | [aA]nti[[:space:]]*[-:][[:space:]]*Pk | anti-Pk |
| anti-Rg | [aA]nti[[:space:]]*[-:][[:space:]]*R(g|ga|g\(a\)|gb|g\(b\)) | anti-Rg, anti-Rga, anti-Rg(a) |
| anti-S | [aA]nti[[:space:]]*[-:][[:space:]]*S[^[:alnum:]] | anti-S |
| anti-s | [aA]nti[[:space:]]*[-:][[:space:]]*s | anti-s |
| anti-Sda | [aA]nti[[:space:]]*[-:][[:space:]]*Sd(a|\(a\)) | anti-Sda, anti-Sd(a) |
| anti-U | [aA]nti[[:space:]]*[-:][[:space:]]*U[^[:alnum:]] | anti-U |
| anti-Ula | [aA]nti[[:space:]]*[-:][[:space:]]*(Ula|K10) | anti-Ula, anti-K10 |
| anti-Vel | [aA]nti[[:space:]]*[-:][[:space:]]*V(E|el) | anti-VE, anti-Vel |
| anti-Wra | [aA]nti[[:space:]]*[-:][[:space:]]*Wr(a|\(a\)) | anti-Wra, anti-Wr(a) |
| anti-Xga | [aA]nti[[:space:]]*[-:][[:space:]]*Xg(a|\(a\)) | anti-Xga, anti-Xg(a) |
| anti-Yka | [aA]nti[[:space:]]*[-:][[:space:]]*Yk(a|\(a\)) | anti-Yka, anti-Yk(a) |
| anti-Yta | [aA]nti[[:space:]]*[-:][[:space:]]*Yt(a|\(a\)) | anti-Yta, anti-Yt(a) |
| anti-Ytb | [aA]nti[[:space:]]*[-:][[:space:]]*Yt(b|\(b\)) | anti-Ytb, anti-Yt(b) |
