# Supplementary material for: Constructing a Population-Based Research Database from Routine Maternal Screening Records: A Resource for Studying Alloimmunization in Pregnant Women
Source: PLoS One. 2011 Nov 30;6(11):e27619. doi: 10.1371/journal.pone.0027619 (PMC3227597; doi:10.1371/journal.pone.0027619)
Supplement: Table S2 — Maternal red blood cell antibodies with a prevalence of less than 1 in10,000 in 920,903 births in Sweden from 1982–2002. (DOCX) [file pone.0027619.s003.docx]

**Table S2**: Maternal red blood cell antibodies with a prevalence of less than 1 in10,000 in 920,903 births in Sweden from 1982-2002.

|  | ***Per 10,000 births*** | | | | |  |  |
| --- | --- | --- | --- | --- | --- | --- | --- |
|  | **1982-1986** | **1987-1991** | **1992-1996** | **1997-2002** | **Overall, 1982-2002** | **Count** | **Percent of  Ab+ births** |
| **Rh system** |  |  |  |  |  |  |  |
| anti-e | 0 | 0.6 | 0.4 | 0.8 | 0.6 | 54 | 0.7 |
| anti-ce | 2.1 | 0.5 | 0.4 | 0.2 | 0.4 | 36 | 0.4 |
| anti-G | 0 | 0 | 0.1 | 0.2 | 0.1 | 9 | 0.1 |
| anti-cE | 0 | 0 | 0.1 | 0 | 0 | 4 | 0 |
| anti-Ce | 0 | 0 | 0 | 0 | 0 | 2 | 0 |
| **MNS system** |  |  |  |  |  |  |  |
| anti-S | 0 | 0.4 | 0.8 | 1.3 | 0.9 | 81 | 1 |
| anti-N | 0 | 0.3 | 0.1 | 0.4 | 0.3 | 25 | 0.3 |
| anti-s | 0 | 0.1 | 0.2 | 0.3 | 0.2 | 18 | 0.2 |
| **Kell system** |  |  |  |  |  |  |  |
| anti-k | 0 | 0.2 | 0.1 | 1.3 | 0.6 | 57 | 0.7 |
| anti-Kpa | 0 | 0.4 | 0.5 | 0.8 | 0.6 | 54 | 0.7 |
| anti-Kpb | 0 | 0 | 0 | 0 | 0 | 2 | 0 |
| **P system** |  |  |  |  |  |  |  |
| anti-PP1Pk | 0 | 0 | 0 | 0.1 | 0 | 5 | 0.1 |
| **Duffy system** |  |  |  |  |  |  |  |
| anti-Fyb | 0 | 0 | 0.3 | 0.2 | 0.2 | 16 | 0.2 |
| **Kidd system** |  |  |  |  |  |  |  |
| anti-Jkb | 0 | 0.1 | 0.2 | 0.5 | 0.3 | 26 | 0.3 |
| **Lutheran system** |  |  |  |  |  |  |  |
| anti-Lub | 0 | 0 | 0 | 0 | 0 | 2 | 0 |
| **Other** |  |  |  |  |  |  |  |
| anti-Ch | 0 | 0 | 0 | 0 | 0 | 2 | 0 |
| anti-Cob | 0 | 0.1 | 0 | 0 | 0 | 5 | 0.1 |
| anti-HI | 0 | 0.1 | 0.6 | 0.3 | 0.3 | 30 | 0.4 |
| anti-I | 0.7 | 0.3 | 0.2 | 0.4 | 0.3 | 28 | 0.3 |
| anti-i | 0.4 | 0 | 0 | 0 | 0 | 1 | 0 |
| anti-IH | 0 | 1.1 | 0.1 | 0 | 0.3 | 26 | 0.3 |
| anti-Jra | 0 | 0 | 0 | 0 | 0 | 1 | 0 |
| anti-Kna | 0 | 0.2 | 0 | 0.2 | 0.1 | 10 | 0.1 |
| anti-LW | 0 | 0 | 0 | 0.1 | 0 | 4 | 0 |
| anti-Sda | 0 | 0.2 | 0.2 | 0.1 | 0.2 | 15 | 0.2 |
| anti-Vel | 0 | 0 | 0 | 0 | 0 | 1 | 0 |
| anti-Wra | 0 | 0 | 0 | 0.1 | 0.1 | 6 | 0.1 |
| anti-Yka | 0 | 0 | 0 | 0 | 0 | 2 | 0 |
| anti-Yta | 0 | 0 | 0.1 | 0.1 | 0 | 5 | 0.1 |
